# Supplementary material for: Characterizing the SASP‐Dependent Paracrine Spreading of Senescence Between Human Brain Cell Types
Source: Aging Cell. 2026 Aug 15;25(8):e70673. doi: 10.1111/acel.70673 (PMC13476760; doi:10.1111/acel.70673)
Supplement: Supplementary file 1 [file ACEL-25-e70673-s001.zip › acel70673-sup-0003-FigureS1-S9.pdf]

Figure S1

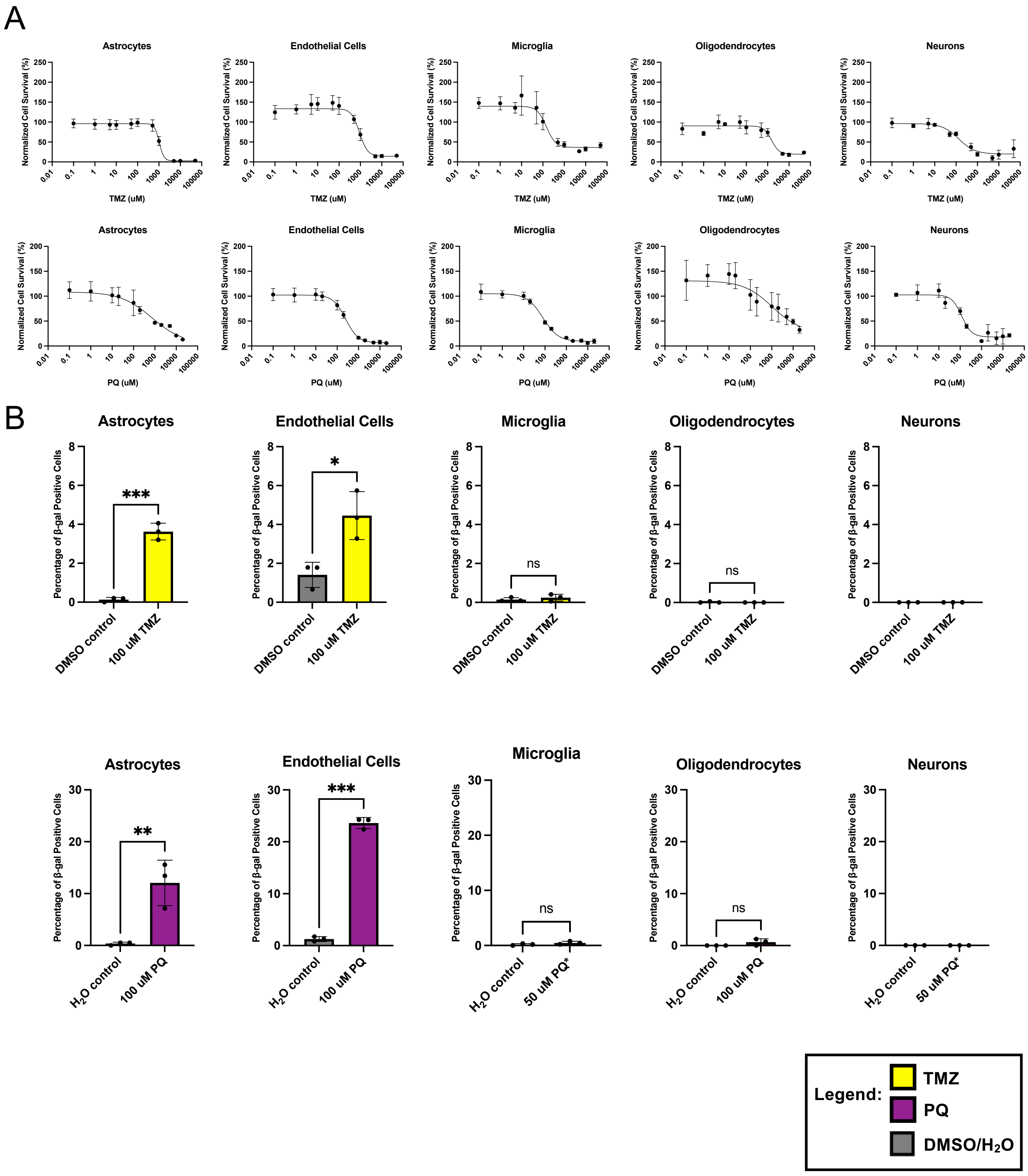
A
Astrocytes
Endothelial Cells
Microglia
Oligodendrocytes
Neurons
Normalized Cell Survival (%)
TMZ (uM)
PQ (uM)
B
Percentage of β-gal Positive Cells
DMSO control
100 uM TMZ
H2O control
100 uM PQ
50 uM PQ*
ns
Legend:
TMZ
PQ
DMSO/H2O

Figure S1. A) Viability curves of human cell lines (astrocytes, endothelial cells, microglia, oligodendrocytes, and neurons) following various concentrations of temozolomide (TMZ) treatment (top) or paraquat (PQ) treatment (bottom) for 48 hours highlighting our selection of treatment with 100 uM TMZ or PQ to attempt to induce senescence (n=3 replicates). Based on increased sensitivity seen of microglia to higher concentrations of PQ, 50 uM treatment was used in this cell type. B) Quantification of percentage of SA β-gal positive cells following 7-day treatment with (top) 100 μM TMZ (yellow) compared to a DMSO control (grey) (n=3 replicates) or (bottom) 100 uM PQ (purple) compared to a H_2O control (grey) (n=3 replicates). Microglia were treated with 50 uM PQ for 7 days due to heightened vulnerability. Data analyzed by unpaired two-tailed t-tests (B). Bar graphs show mean with error bars depicting standard deviation (ns, $p>0.05$, * $p<0.05$, ** $p<0.01$, *** $p<0.001$).

Figure S2

A

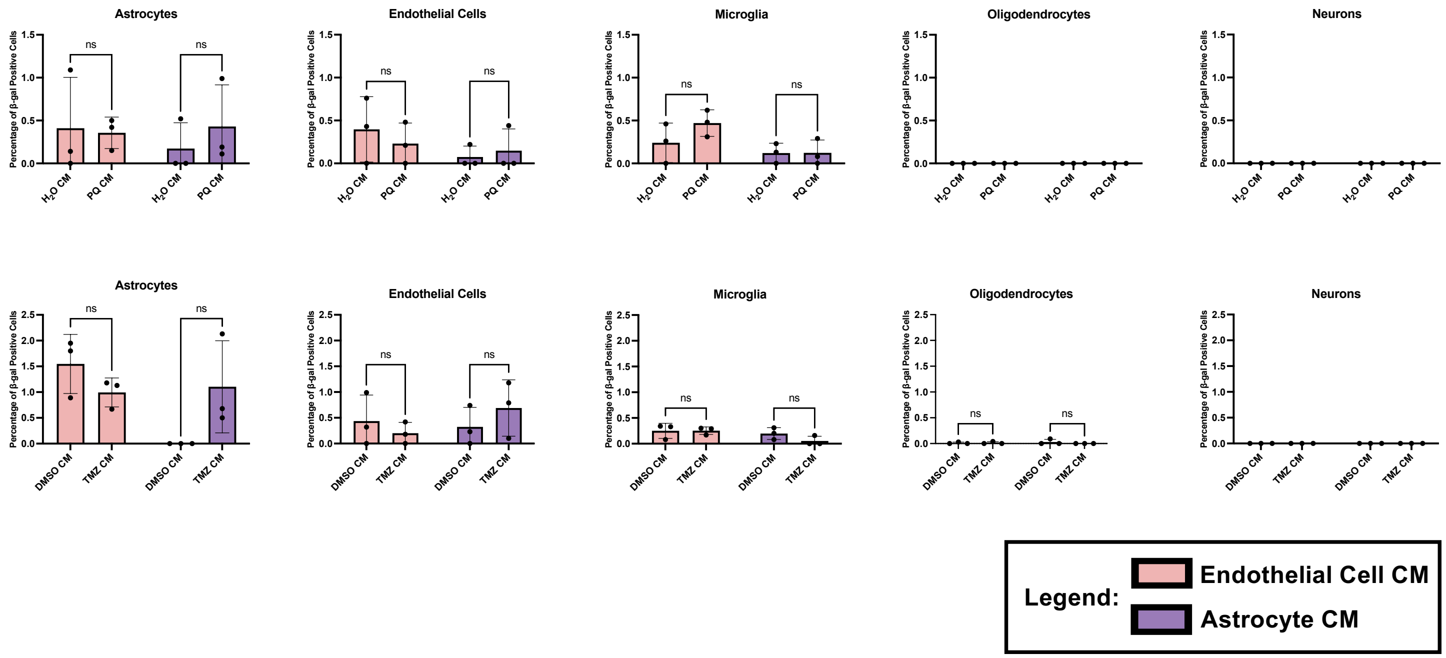
Astrocytes
Endothelial Cells
Microglia
Oligodendrocytes
Neurons
Percentage of β-gal Positive Cells
ns
H2O CM
PQ CM
DMSO CM
TMZ CM
Legend:
Endothelial Cell CM
Astrocyte CM

Figure S2. A) Quantification of percentage of SA β-gal positive senescent astrocytes, endothelial cells, microglia, oligodendrocytes, and neurons following 7-day treatment with endothelial cell (pink) and astrocyte (purple) DMSO and TMZ CM as well as H_2O and PQ CM (n=3 replicates). Data analyzed by two-way ANOVA with Tukey's multiple comparisons test. All graphs show mean with error bars depicting standard deviation (ns, $p>0.05$).

Figure S3

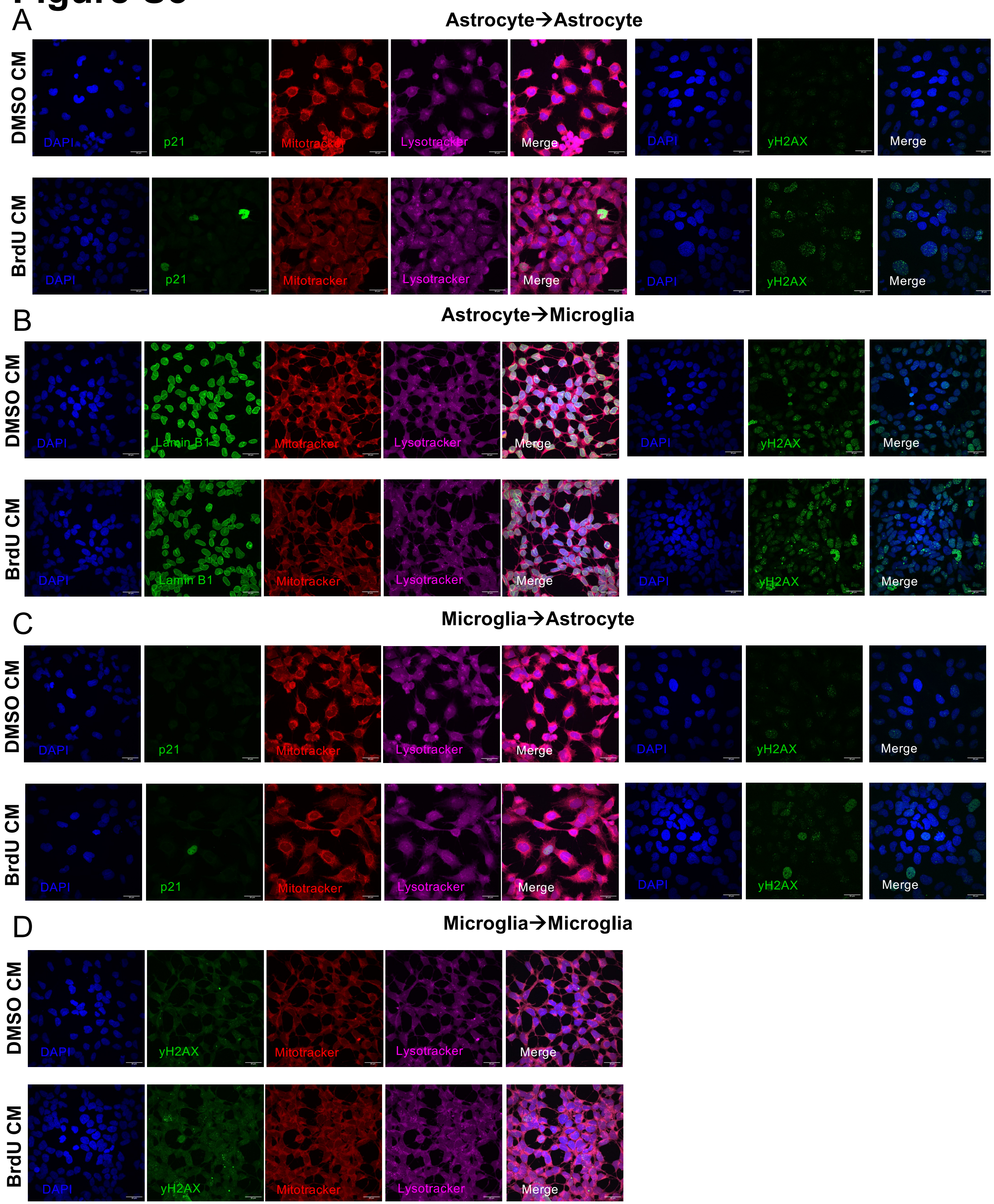
A
Astrocyte→Astrocyte
DMSO CM
BrdU CM
DAPI
p21
Mitotracker
Lysotracker
Merge
yH2AX
B
Astrocyte→Microglia
Lamin B1
C
Microglia→Astrocyte
D
Microglia→Microglia

Figure S3. A) Confocal images of astrocytes treated with DMSO CM and BrdU CM from astrocytes with staining for p21 (green), Mitotracker Red CMXRos (red), Lysotracker (purple) and yH2AX (green) with DAPI (blue). B) Confocal images of microglia treated with DMSO CM and BrdU CM from astrocytes with staining for lamin B1 (green), Mitotracker Red CMXRos (red), Lysotracker (purple) and yH2AX (green) with DAPI (blue). C) Confocal images of astrocytes treated with DMSO CM and BrdU CM from microglia with staining for p21 (green), Mitotracker Red CMXRos (red), Lysotracker (purple) and yH2AX (green) with DAPI (blue). D) Confocal images of microglia treated with DMSO CM and BrdU CM from microglia with staining for yH2AX (green), Mitotracker Red CMXRos (red), Lysotracker (purple) with DAPI (blue). All scale bars are 30 μm.

Figure S4

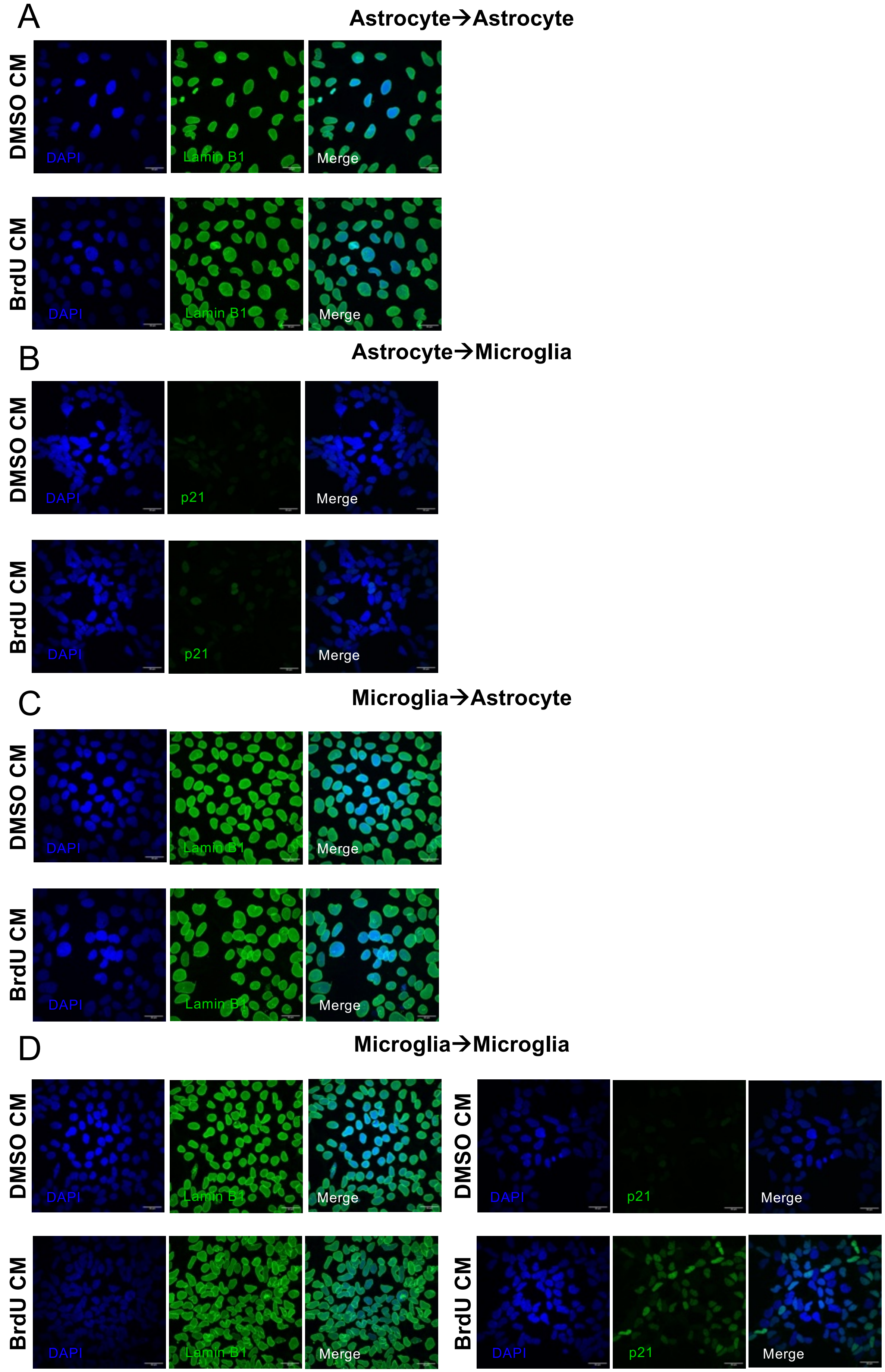
A
Astrocyte→Astrocyte
DMSO CM
BrdU CM
DAPI
Lamin B1
Merge
B
Astrocyte→Microglia
p21
C
Microglia→Astrocyte
D
Microglia→Microglia

Figure S4. A) Confocal images of astrocytes treated with DMSO CM and BrdU CM from astrocytes with staining for lamin B1 (green) with DAPI (blue). B) Confocal images of microglia treated with DMSO CM and BrdU CM from astrocytes with staining for p21 (green) with DAPI (blue). C) Confocal images of astrocytes treated with DMSO CM and BrdU CM from microglia with staining for lamin B1 (green) with DAPI (blue). D) Confocal images of microglia treated with DMSO CM and BrdU CM from microglia with staining for lamin B1 (green) with DAPI (blue) and p21 (green) with DAPI (blue). All scale bars are 30 μm.

Figure S5

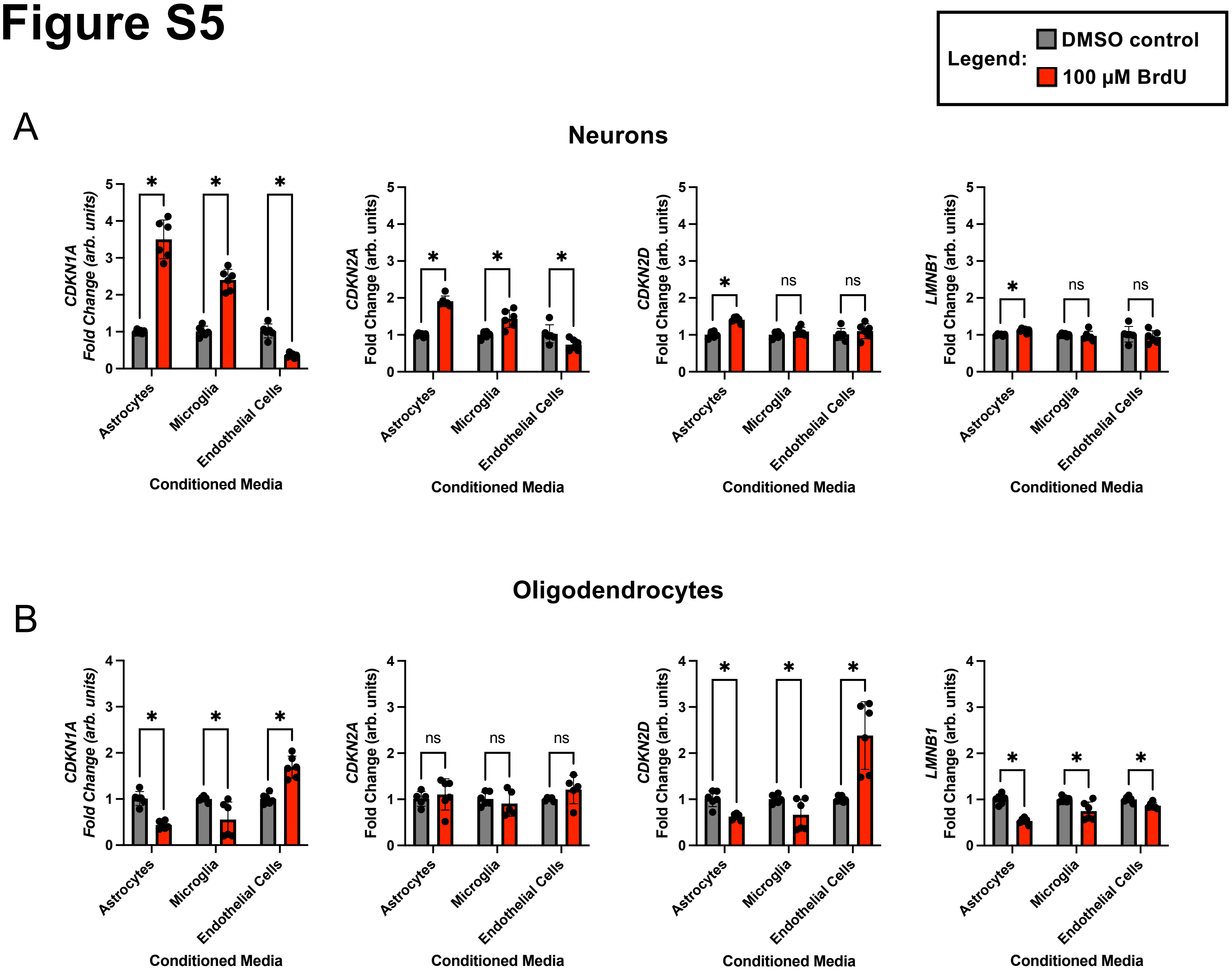
Legend:
DMSO control
100 µM BrdU
A
Neurons
B
Oligodendrocytes
CDKN1A Fold Change (arb. units)
CDKN2A Fold Change (arb. units)
CDKN2D Fold Change (arb. units)
LMNB1 Fold Change (arb. units)
Astrocytes
Microglia
Endothelial Cells
Conditioned Media
*
ns

Figure S5. A) Quantitative PCR of astrocyte, microglia, and endothelial cell DMSO CM (grey) and 100 µM BrdU CM (red) treated neurons for *CDKN1A*, *CDKN2A*, *CDKN2D*, and *LMNB1* (n=6 replicates). B) Quantitative PCR of astrocyte, microglia, and endothelial cell DMSO CM (grey) and 100 µM BrdU CM (red) treated oligodendrocytes for *CDKN1A*, *CDKN2A*, *CDKN2D*, and *LMNB1* (n=6 replicates). Data analyzed by unpaired two-tailed t-test (A-B). All graphs show mean with error bars depicting standard deviation (ns, $p>0.05$, * $p<0.05$).

Figure S6

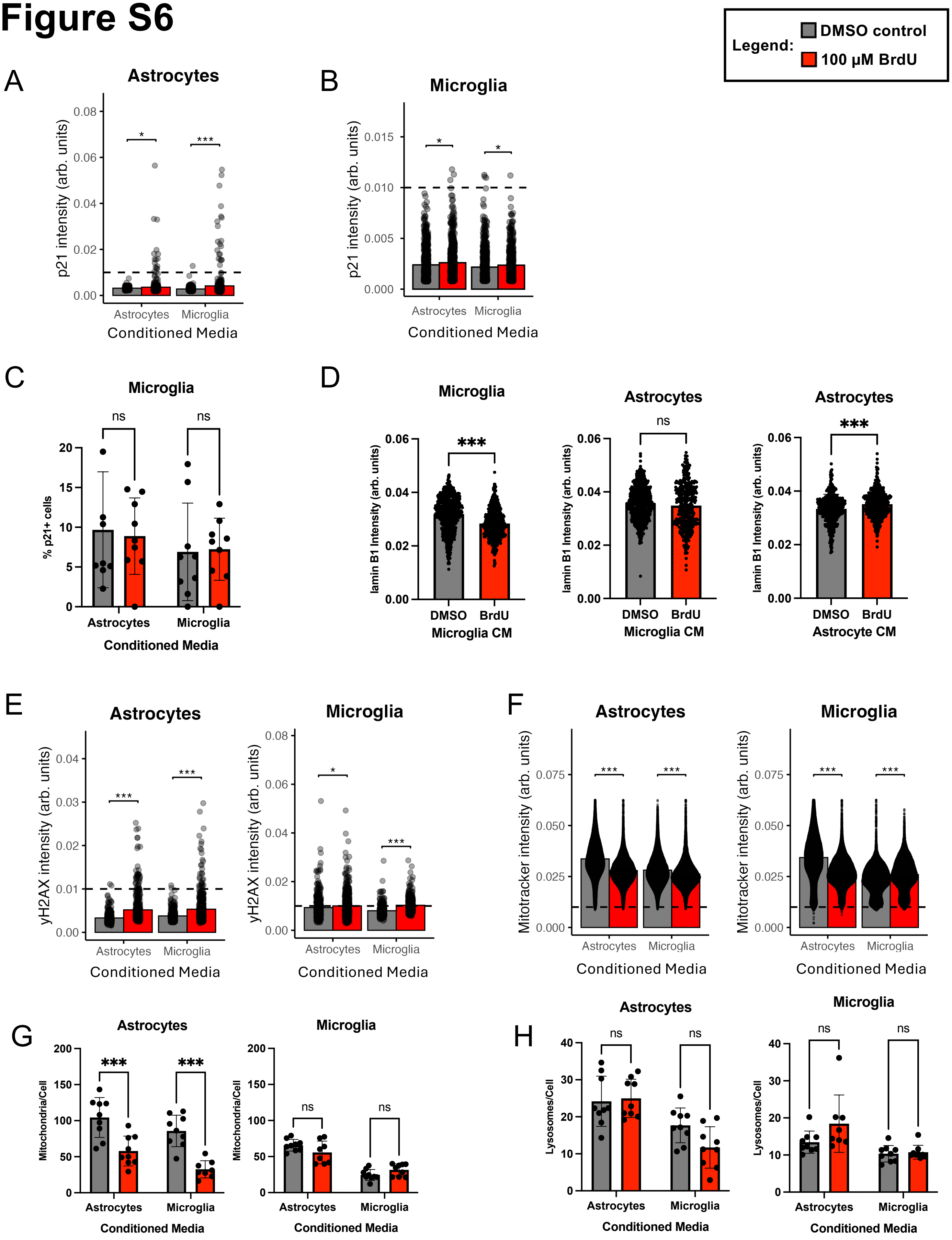

Legend:
DMSO control
100 µM BrdU
A
Astrocytes
p21 intensity (arb. units)
Conditioned Media
Astrocytes
Microglia
B
Microglia
C
Microglia
% p21+ cells
ns
D
Microglia
Astrocytes
lamin B1 Intensity (arb. units)
DMSO
BrdU
Microglia CM
Astrocyte CM
E
yH2AX intensity (arb. units)
F
Mitotracker intensity (arb. units)
G
Mitochondria/Cell
H
Lysosomes/Cell

Figure S6. Quantification of p21 intensity in astrocytes (A) and microglia (B) treated with DMSO CM (grey) and BrdU CM (red) from astrocytes and microglia (n=9 replicates). C) Quantification of percentage (%) of p21 positive cells in microglia treated with DMSO CM and BrdU CM from astrocytes and microglia (n=9 replicates). D) Quantification of lamin B1 intensity in microglia treated with DMSO CM (grey) and BrdU CM (red) from microglia (left), astrocytes treated with DMSO CM (grey) and BrdU CM (red) from microglia (middle), and astrocytes treated with DMSO CM (grey) and BrdU CM (red) from astrocytes (right) (n=9 replicates). E) Quantification of γH2AX intensity in astrocytes (left) and microglia (right) treated with DMSO CM (grey) and BrdU CM (red) from astrocytes and microglia (n=9 replicates). F) Quantification of Mitotracker Red CMXRos intensity in astrocytes (left) and microglia (right) treated with DMSO CM (grey) and BrdU CM (red) from astrocytes and microglia (n=9 replicates). G) Quantification of mitochondrial mass (mitochondria/cell) based on Mitotracker Red CMXRos staining in astrocytes (left) and microglia (right) treated with DMSO CM (grey) and BrdU CM (red) from astrocytes and microglia (n=9 replicates). H) Quantification of lysosomal mass (lysosomes/cell) based on Lysotracker staining in astrocytes (left) and microglia (right) treated with DMSO CM (grey) and BrdU CM (red) from astrocytes and microglia (n=9 replicates). Data was analyzed by two-way ANOVA with Tukey's multiple comparisons test (A-C, E-H) and unpaired Mann-Whitney t-test (D). All graphs show mean with error bars depicting standard deviation (ns, $p>0.05$, * $p<0.05$, *** $p<0.001$).

Figure S7

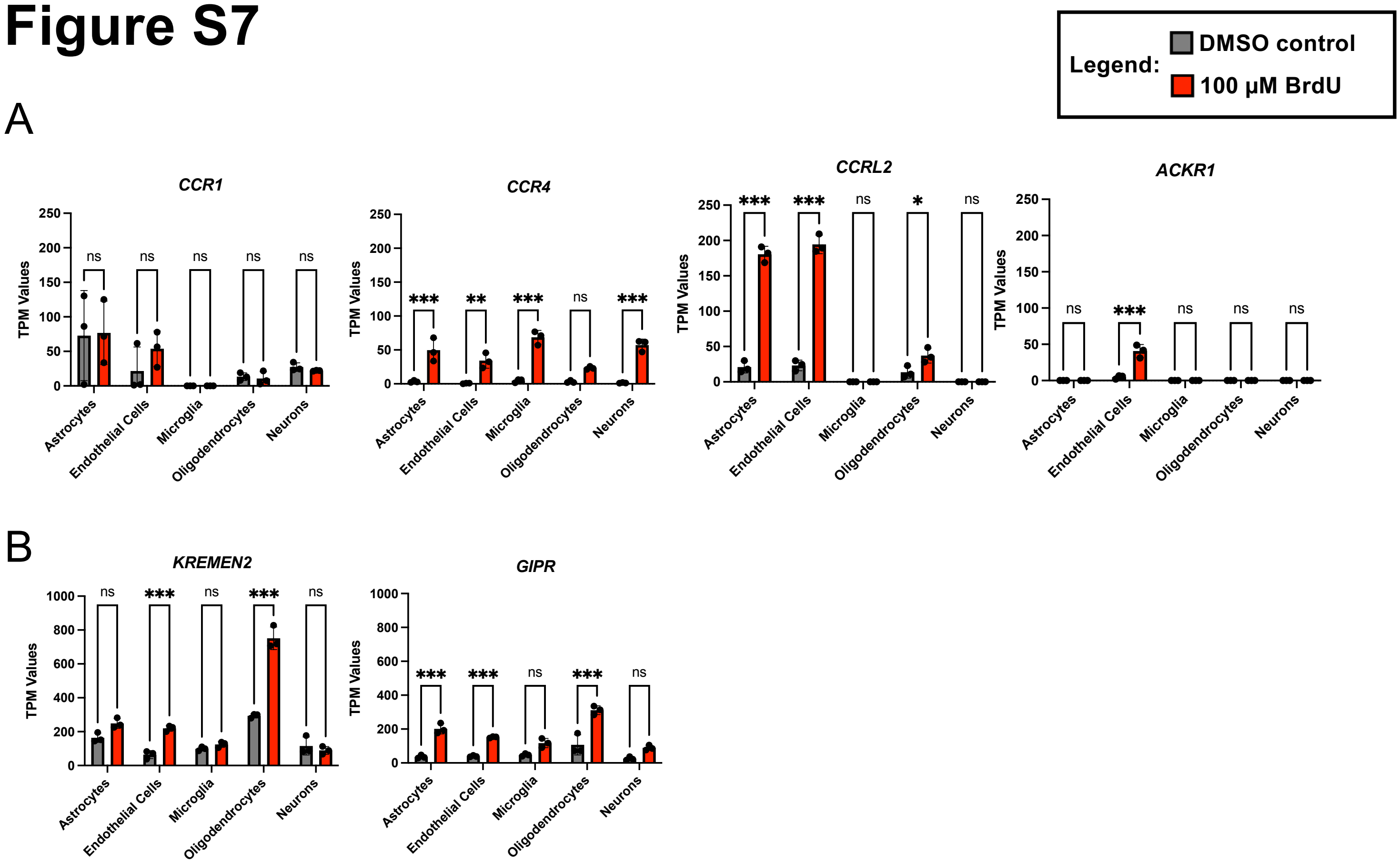
Legend:
DMSO control
100 μM BrdU
A
CCR1
CCR4
CCRL2
ACKR1
TPM Values
Astrocytes
Endothelial Cells
Microglia
Oligodendrocytes
Neurons
ns
***
**
*
B
KREMEN2
GIPR

Figure S7. A) TPM expression values of CCL2 receptors *CCR1, CCR4, CCRL2,* and *ACKR1* in DMSO (grey) and BrdU (red) treated cell lines (n=3 replicates). B) TPM expression values of receptors *KREMEN2* and GIPR in DMSO (grey) and BrdU (red) treated cell lines (n=3 replicates). Data was analyzed by two-way ANOVA with Tukey's multiple comparisons test (A-B). All graphs show mean with error bars depicting standard deviation (ns, $p>0.05$, * $p<0.05$, ** $p<0.01$, *** $p<0.001$).

Figure S8

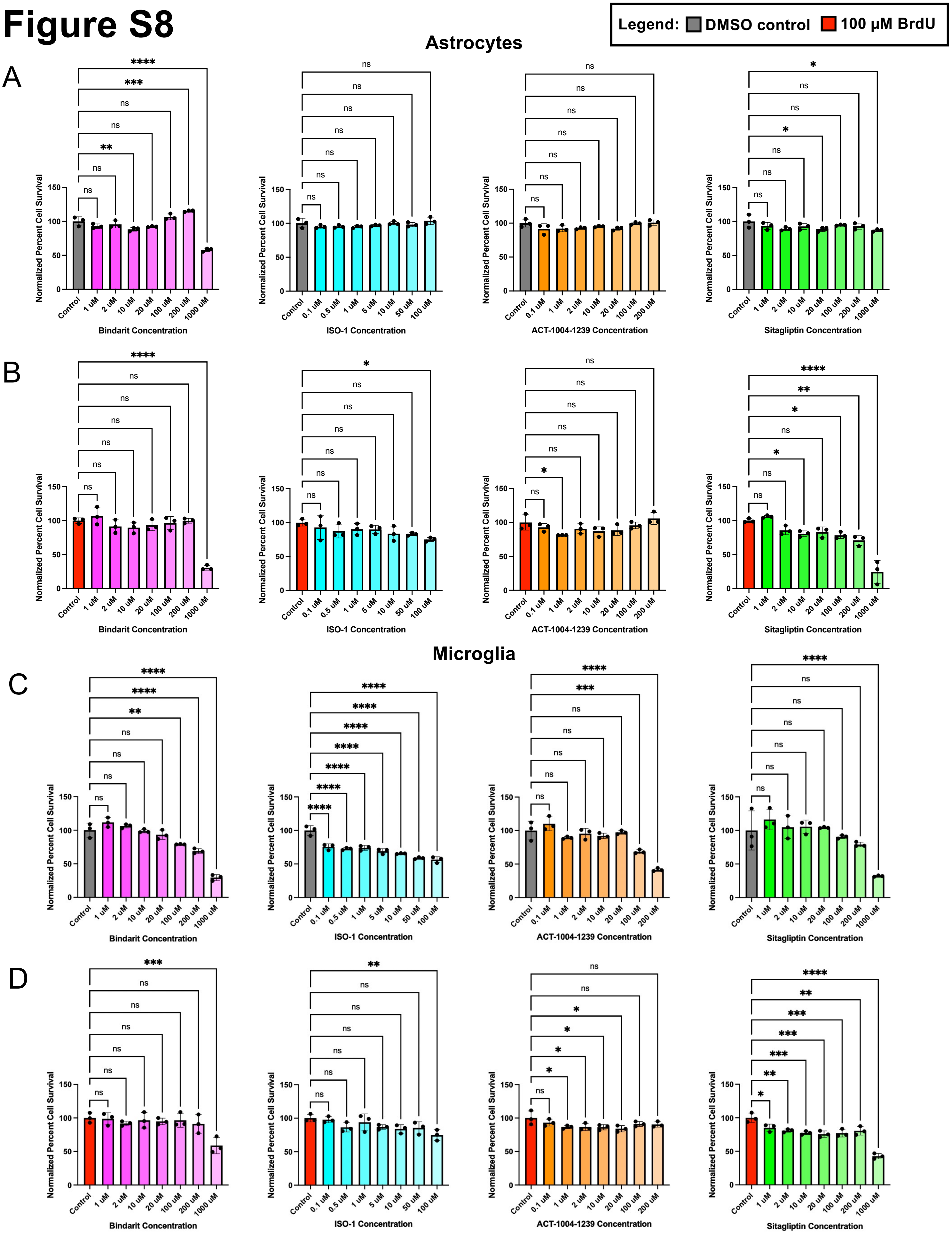

Legend: DMSO control 100 µM BrdU
Astrocytes
A
B
Microglia
C
D
Normalized Percent Cell Survival
Control
1 uM
2 uM
10 uM
20 uM
100 uM
200 uM
1000 uM
0.1 uM
0.5 uM
5 uM
50 uM
Bindarit Concentration
ISO-1 Concentration
ACT-1004-1239 Concentration
Sitagliptin Concentration
ns
*
**
***
****

Figure S8. A) CCK8 viability assay in astrocytes showing normalized percent cell survival following treatment with DMSO control (grey) or DMSO + Bindarit (pink), DMSO + ISO-1 (blue), DMSO + ACT-1004-1239 (orange), or DMSO + Sitagliptin (green) at various concentrations (n=3 replicates). B) CCK8 viability assay in astrocytes showing normalized percent cell survival following treatment with 100 μM BrdU (red) or BrdU + Bindarit (pink), BrdU + ISO-1 (blue), BrdU + ACT-1004-1239 (orange), or BrdU + Sitagliptin (green) at various concentrations (n=3 replicates). C) CCK8 viability assay in microglia showing normalized percent cell survival following treatment with DMSO control (grey) or DMSO + Bindarit (pink), DMSO + ISO-1 (blue), DMSO + ACT-1004-1239 (orange), or DMSO + Sitagliptin (green) at various concentrations (n=3 replicates). D) CCK8 viability assay in microglia showing normalized percent cell survival following treatment with 100 μM BrdU (red) or BrdU + Bindarit (pink), BrdU + ISO-1 (blue), BrdU + ACT-1004-1239 (orange), or BrdU + Sitagliptin (green) at various concentrations (n=3 replicates). Data analyzed by one-way ANOVA with Dunnett's correction for multiple comparisons (A-D). All graphs show mean with error bars depicting standard deviation (ns, $p>0.05$, * $p<0.05$, ** $p<0.01$, *** $p<0.001$).

Figure S9

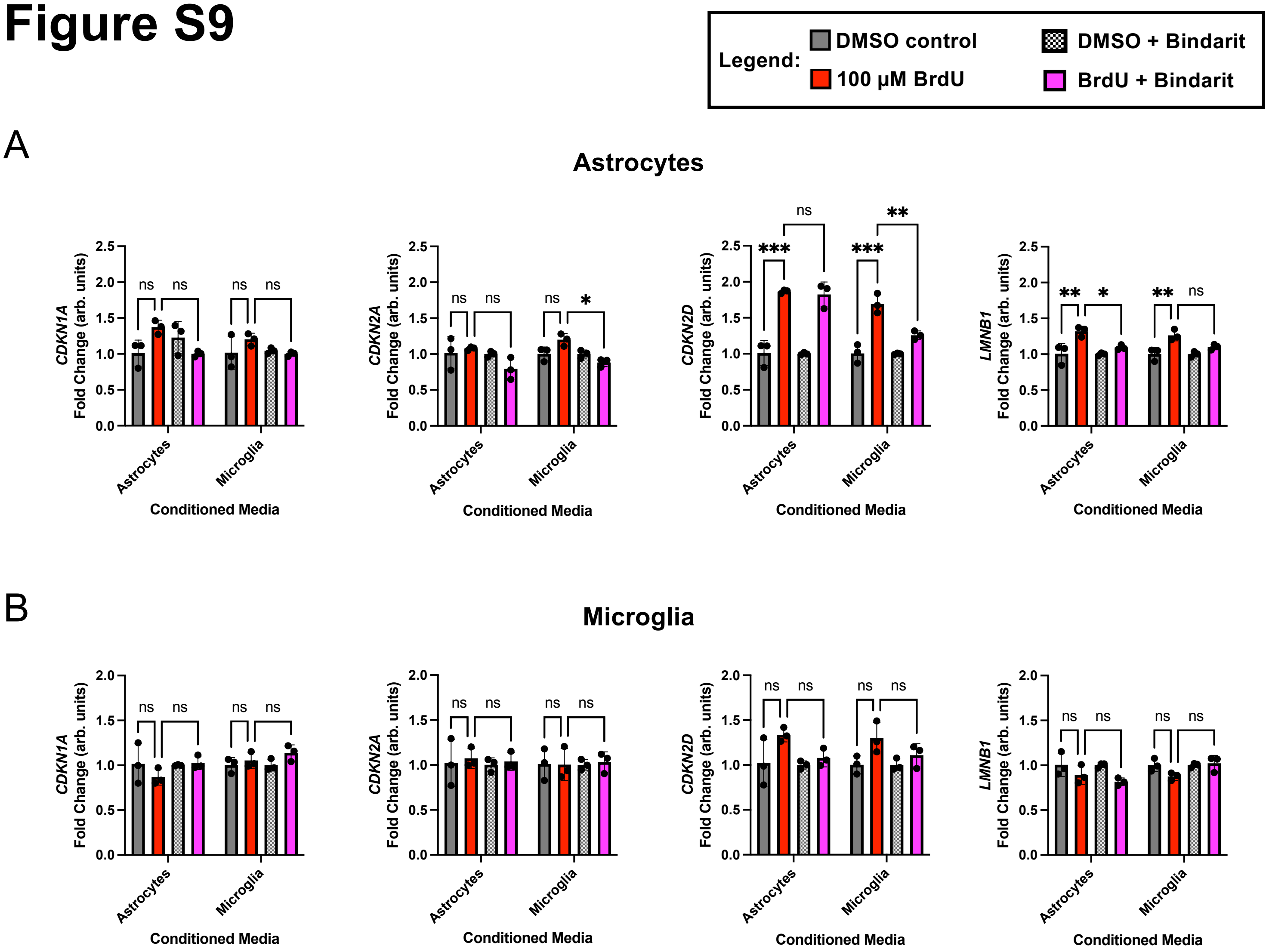

Legend:
DMSO control
100 µM BrdU
DMSO + Bindarit
BrdU + Bindarit
A
Astrocytes
CDKN1A Fold Change (arb. units)
CDKN2A Fold Change (arb. units)
CDKN2D Fold Change (arb. units)
LMNB1 Fold Change (arb. units)
ns
*
**
***
Astrocytes
Microglia
Conditioned Media
B
Microglia
CDKN1A Fold Change (arb. units)
CDKN2A Fold Change (arb. units)
CDKN2D Fold Change (arb. units)
LMNB1 Fold Change (arb. units)
ns
Astrocytes
Microglia
Conditioned Media

Figure S9. A) Quantitative PCR of astrocytes treated with DMSO CM (grey), BrdU CM (red), DMSO + Bindarit CM (checkered), or BrdU + Bindarit CM (purple) from both astrocytes and microglia for *CDKN1A*, *CDKN2A*, *CDKN2D*, and *LMNB1* (n=3 replicates). B) Quantitative PCR of astrocytes treated with DMSO CM (grey), BrdU CM (red), DMSO + Bindarit CM (checkered), or BrdU + Bindarit CM (purple) from both astrocytes and microglia for *CDKN1A*, *CDKN2A*, *CDKN2D*, and *LMNB1* (n=3 replicates). Data analyzed by two-way ANOVA with Šídák's multiple comparisons test (A-B). All graphs show mean with error bars depicting standard deviation (ns, $p>0.05$, * $p<0.05$, ** $p<0.01$, *** $p<0.001$).
